# Reasons for high prevalence of contraceptive withdrawal in Tehran, Iran

**DOI:** 10.1038/s41598-023-37398-1

**Published:** 2023-06-29

**Authors:** Amir Erfani, Ali-Asghar Kolahi

**Affiliations:** 1 Department of Sociology and Anthropology, Nipissing University, 100 College Drive, Box 5002, North Bay, ON P1B 8L7 Canada; 2 Social Determinants of Health Research Center, Shahid Beheshti University of Medical Sciences, Tehran, Iran

**Keywords:** Population screening, Health policy

## Abstract

This study explores reasons for withdrawal use that is highly prevalent in Iran. A face-to-face semi-structured survey questionnaire was designed and 79 married women aged 15–49, who were only using withdrawal when attending five primary healthcare centers in Tehran during September–October 2021 were interviewed. Results showed that withdrawal mostly was chosen by the couple (67%), and partly by the woman alone (19%) or by the husband alone (14%). Participants evaluated withdrawal positively that has no side effect and cost, is easy to use and accessible, and increases sexual pleasure and intimacy. Most women agreed that husbands use withdrawal to protect their wife's health (76%). Women obtained contraceptive information primarily from gynecologists (42%), the internet (21%), midwives in public health centers (19%), and social networks (18%). "Side effects of modern methods" (37%), "fear of side effects" (16%), and "reduction in sexual pleasure" (14%) were the major reasons reported for using withdrawal. While 'side effects' was given mostly by women who alone or with their husband chose withdrawal (52%, 38%), 'reduction in sexual pleasure' and 'fear of side effects' were mostly reported by women whose husband was the sole decision maker in choosing withdrawal (28%, 25%). The 'fear of side effects' was reported mostly by women who had lower education (21%), used the internet for contraceptive information (23%), and whose husband alone chose withdrawal (25%). Cost of modern methods was a trivial reason for using withdrawal. Most withdrawal users (75%) would not switch to modern methods even if they were freely accessible. More educated women and their husbands would be less likely to switch to modern methods even they were provided freely (OR 0.28, CI 0.10–0.80; OR 0.20, CI 0.07–0.59). However, women who were using modern methods before, and those who alone chose withdrawal would be more likely to switch to modern methods (OR 6.4, CI 2.0–20.2; OR 3.4, CI 1.1–11.2). Access to regular contraceptive counselling and public health campaigns could help women to deal with fears of side effects of modern methods, learn their proper use, and to receive education on how to use withdrawal more effectively to avoid unintended pregnancies.

Withdrawal (coitus interruptus or pulling out), as the male traditional contraceptive method, is one of the oldest temporary contraceptive methods used in the world^1^. It is estimated that 5.5% of all contraceptive users, equated to 53 million women of reproductive age, use withdrawal as their primary contraceptive method worldwide^2^. This method is especially common in certain countries in Eastern Europe, Central and Western Asia, including Iran.

Along with induced abortion, withdrawal has played an important role in the demographic transition of Western countries and Islamic societies, including Iran^1,3–6^. Moreover, as an "ultramodern contraception" with no hormonal side effects that men can participate in and is always freely available to couples without requiring interactions with healthcare providers, withdrawal has been prevalent among more educated, wealthier women living in urban settings in India and Iran and other places^7–9^. Despite these advantages, withdrawal has a relatively high typical-use failure rate, (Typical-use withdrawal failure rate refers to the withdrawal failure among all women using withdrawal, including those who use it inconsistently and incorrectly.) estimated at 20% for the United States and 17.3% for 15 developing countries with quality data, which vary significantly by country, age, contraceptive intention and marital status^10,11^. For Iran, the average typical-use failure rate of withdrawal was estimated at 12%, which varies from 21% among women under the age of 25 to 8% among women aged 30 or higher^12^. The large variations in failure rates by age are due to the facts that with increasing women's age, their biological fecundity and coital frequency decrease and their contraceptive experience increases^13,14^.

The high prevalence of withdrawal in certain Eastern European and Asian countries, including Iran, remains a concern for stakeholders in reproductive health and population policy, as its relatively high typical-use failure rate has led to more than half of unwanted pregnancies and induced abortions in Asian countries^11,15–17^. This concern is particularly pronounced in Iran, where abortion is legally highly restricted and permitted only in case of fetal impairment, and one-fifth of pregnancies remain unintended^15^. It is estimated that annually about 129,000 clandestine abortions occur outside the legal system^18,19^, likely with adverse health risks and socioeconomic burdens for the individual and society. The results of fertility surveys conducted in the capital city of Iran, containing one-seventh of the national population, revealed that 48% of unintended pregnancies (mistimed plus unwanted) in the five years preceding the survey resulted from failures of withdrawal, 20 percent from failures in modern contraceptive use, 26% contraceptive discontinuation, and 6 percent from contraceptive non-use^15^. In addition, failure in the use of withdrawal accounted for nearly half of pregnancies (48%) aborted in the six years preceding the 2009 and 2014 fertility surveys^16^. Moreover, the unmet need for modern contraceptive methods in Iran is estimated to be 17.4%^20^. These adverse fertility outcomes are expected to rise, as the proportion of withdrawal users have been increasing over the past decades in Iran.

The use of withdrawal has increased from 17.8% in 2000 to 21% in 2010 among married women of reproductive age in Iran^21,22^. The rise in male contraceptive methods has been sharper in Tehran. The use of withdrawal and male condoms respectively increased from 28% and 6% in 2000 to 34% and 21%, while the proportion of Intrauterine Device (IUD) and Pill users dropped from 15% and 12% in 2000 to 7% each in 2014 in Tehran^22^. The increasing trend of withdrawal use happened during the period that modern contraceptive methods were freely accessible in the public rural health units and urban health centers, suggesting that even access to free contraceptive methods did not deter women from using withdrawal. Therefore, there should be reasons for preferring withdrawal to modern methods that need to be investigated.

Following the country's enduring below-replacement fertility rate, the State officially removed the old family planning program in 2017 with the hope to encourage childbearing and increase fertility rates^23^. Since then, however, the total fertility rate has not increased and has been hovering around 1.7 children per woman^24^. In fact, the government incorrectly gave too much credit to the fertility-inhibiting effect of the publicly funded family planning services. Prior to the removal of the family planning program, only 22% of married women aged 15–49 attended public health units to receive family planning services. Others mainly used male contraceptives of withdrawal and condoms^25^. Thus, based on evidence from Tehran, only 7% of the reduction in fertility in 2014 was attributed to the effective use of medical sterilization and IUDs, as two major contraceptive methods subsidised by the government. The other 93% of the fertility reduction was caused by delaying marriage, use of withdrawal, condoms, oral contraceptives (the pill), induced abortion and postpartum infecundability, "all of which have less to do with the government’s family planning programme." Pills and condoms are accessible in supermarkets and drugstores with affordable cost, and withdrawal is always accessible with no charge^25^. Therefore, the limitation on the provision of publicly funded family planning services largely will affect women of low socioeconomic status, if they decide to use long-acting methods, namely IUDS^23^.

Although all public health units and hospitals are currently banned to distribute contraceptive services and methods, family healthcare providers in the public health units only can provide on-demand contraceptive knowledge counselling, geared toward healthy fertility rather than limiting births^23^. Therefore, women requiring contraceptive methods and services should refer to private sectors, including pharmacies and private practice gynecologists and midwives. One of the objectives of this study is to explore to what extent the likely "cost of modern contraceptive methods" can be the reason for choosing to use withdrawal.

Apart from this likely economic reason, it is not yet clear why women who have easy access to Pills, Condoms and IUDs through private sectors, still prefer to use withdrawal, which is often regarded as a less effective contraceptive by service providers. The limited number of studies documenting the determinants of withdrawal use have focused mostly on demographic characteristics of women and have provided conflicting findings, with superficial explanations. For example, a recent study on withdrawal use in Albania, Armenia, Jordan, and Turkey show that socio-demographic determinants varied by country, though the general trends indicated that with increasing parity, education, and household wealth women were less likely to use withdrawal than modern methods^26^. Evidence from analyzing 80 national surveys showed that traditional contraceptive methods, including withdrawal, are more widespread among young, sexually active unmarried women than their married counterparts^27^. Moreover, the findings from Iran show that withdrawal users are generally younger, well educated, wealthier, and from urban areas^28,29^. By contrast, evidence indicates that withdrawal users in Turkey are largely women with lower levels of education and wealth who live in rural areas, with a less-egalitarian gender relationship^30,31^. When it comes to explaining why women with the observed characteristics prefer withdrawal to modern methods, the studies fail to provide evidence-based explanations, and instead suggest some speculations. In fact, couples' reasons for choosing a contraceptive method are directly related to their contraceptive knowledge, evaluation and attitudes, and availability^32^. There are limited studies examining these direct factors of contraceptive choice. Earlier studies conducted before the removal of the family planning program in Iran have provided limited insights on the reasons for withdrawal use. They have suggested that couples prefer withdrawal to other methods, as it has no adverse effects and cost, is accessible and easy to use, does not require healthcare visits, and is "natural"^33,34^. However, the information about reasons for withdrawal and their correlates after the recent removal of the public family program is limited. This study aims to address this gap by exploring the reasons for not using modern contraceptive methods instead of withdrawal, and examine women's source of contraceptive knowledge, attitudes and evaluation of contraceptive withdrawal, and their willingness to switch to modern methods if they are accessible. The results of this descriptive pilot study are expected to expand our understanding of the rationales behind choosing withdrawal method in Iran and elsewhere.

## Methods

### Study design

To address the objectives of this study, a cross-sectional pilot survey was designed and implemented in a sample of 100 married women of reproductive age (15–49) during September–November 2021. The study participants were selected randomly from the clients who attended five health centers (as training centers affiliated to Shahid Beheshti University of Medical Sciences) located in the north, east, and southeast districts of the city of Tehran, the capital city of Iran. Ten midwives working in the five health centers were hired and trained to conduct the face-to-face interviews (each 10 interviews) with eligible women at certain days of a week selected randomly. An eligible client (woman) who approached a midwife in the selected public health center to receive health services was invited to participate in the interview. Criteria for inclusion were married women, aged 15–49, withdrawal users at the time of the interview, and sexually active. Out of 100 completed interviews, 21 women used withdrawal with male condoms or pills. These dual-method users were excluded from the study, as condoms and pills are 'modern methods', and the failure rate of combined methods is lower than that of withdrawal. Therefore, the study sample was limited to 79 eligible women who used only withdrawal. Generally, residences in the northern, eastern, and southeastern residential districts of Tehran are respectively from rich, moderate, and poor wealth status. Therefore, this study includes participants from different wealth backgrounds, though education levels of women and their husbands will be used in all analyses to better represent the individual's socioeconomic status.

A semi-structured survey questionnaire was designed that included 43 close-ended and open-ended questions. The survey included questions about participants' socio-demographic characteristics, current contraceptive use, reasons for not using modern methods, reasons for switching contraceptive methods, reasons for using withdrawal versus condoms as the other male method, evaluation of advantages and disadvantages of withdrawal, willingness to switch to modern methods, sources of contraceptive information, and attitudes toward withdrawal method. The attitude items were developed based on information obtained from explorative interviews the first author conducted with midwives.

### Analysis

Descriptive analyses were conducted to describe the participants' characteristics, their source of contraceptive information, evaluation of and attitudes towards withdrawal, reasons for withdrawal use, and willingness to switch to modern methods. Bivariate analyses, Chi-square test and unadjusted odds ratio from binary logistic regression, were utilized to examine whether observed sociodemographic differences in the reasons for using withdrawal, willingness to switch to modern methods, source of contraceptive information and attitudes towards withdrawal are statistically significant at the *p*-value ≤ 0.05. Specifically, differences in reasons for withdrawal use according to past contraceptive use, source of contraceptive information, and gender role in choosing withdrawal were examined. Univariate binary logistic regression was employed to estimate odds of willingness to switching from withdrawal to other contraceptive methods on the condition that they are provided freely by selected covariates, including past contraceptive use and gender role in choosing withdrawal. All analyses were carried out by SPSS version 27.0.

### Ethics

Nipissing University in Canada (File Number: 102748) and Shahid Beheshti University of Medical Sciences in Iran (Approval ID: IR.SBMU.RETECH.REC.1400.277) provided ethics approvals for the study. Informed consent was obtained from all participants before the interview. Participants voluntarily participated in the study and did not receive any compensations for the interview. They had the right to withdraw from the interview at any time if they wished to do so. They were assured that their answers would remain anonymous. All interviews were performed in accordance with relevant guidelines and regulations.

## Results

### Characteristics of participants

Table 1 shows that the mean age of participants and their husbands were respectively 34.9 (SD = 6.2) and 39.3 (SD = 6.6) years. Participants' age ranged from 21 to 47 years. About half (47%) of participants were married for less than 10 years, with the mean duration of 11.0 (SD = 6.3) years. Participants and their husbands completed 14.7 (SD = 3.8) and 14.6 (SD = 3.6) years of schooling, respectively. More than one-third (33%) of women were employed, and 60% reported to have a Fars ethnic background. On average, women had 1.8 (SD = 1.1) pregnancies and 1.4 (SD = 0.8) living children. Nearly three out of five women (61%) reported that they did not intend to have (another) children. About three out of four (70%) women reported that they "always" "easily talk to their husband about choosing a contraceptive method". While 19% of participants (women) reported that "it was more my preference than my husband's" to use the withdrawal method, 14% reported that it was mostly their husband's preference, and the rest of participants (67%) stated that they both (husband and wife) preferred the withdrawal method. The mean duration of withdrawal use was 8.1 (SD = 5.8) years, and only 23% of participants used a modern contraceptive method before the current use of withdrawal. They switched either from condoms, pills or IUD to withdrawal.

**Table 1:** Percentage distribution of Withdrawal users (married women aged 15–49 years), according to selected characteristics: Tehran, Iran (n = 79).

Characteristics	Percent	Mean (SD)
Women's age (years)		34.9 (6.2)
< 35	46.8	
≥ 35	53.2	
Husband's age (years)		39.3 (6.6)
< 40	53.2	
≥ 40	46.8	
Marriage duration (years)		11.0 (6.3)
< 10	46.8	
≥ 10	53.2	
Women's years of schooling		14.7 (3.8)
≤ 12	32.9	
12 +	67.1	
Husband's years of schooling		14.6 (3.6)
≤ 12	36.7	
12 +	63.3	
Employment status		
Unemployed	67.1	
Employed	32.9	
Ethnicity		
Fars	59.5	
Else (Turk & others)	40.5	
No. of pregnancies		1.8 (1.1)
< 2	38.0	
≥ 2	62.0	
No. of children		1.4 (0.8)
< 2	51.9	
≥ 2	48.1	
Fertility intention		
Don't want children	60.8	
Want children/unsure	39.2	
How often easily do you talk to your husband about choosing a contraceptive method?		
Always	69.6	
Sometimes/rarely/never	30.4	
Who mostly prefers to use withdrawal?		
Wife	19.0	
Husband	13.9	
Both	67.1	
Duration of withdrawal use (years)	–	8.8 (5.9)
Before the withdrawal method, what contraceptive method did you use?		
No other methods, only withdrawal	53.2	
Did not use any contraceptive methods	24.0	
Modern methods*	22.8	

SD,  Standard Deviation. * Pills (n = 8), condoms (n = 8), IUD (n = 2).

### Source of contraceptive information

When participants were asked "When you have a problem or question about contraceptive methods, who do you usually talk to first and get information?", they named "gynecologists" (42%) and "the internet" (21%) as their first two primary sources of contraceptive knowledge. Other sources included "midwife in the health centers" (19%) and "Social networks", including relatives, friends and colleagues (18%). Further bivariate analyses in Table 2 indicated that women who were aged 35 or older (48%), employed (54%), and had some postsecondary schooling (45%), did not intend to have children (46%), and occasionally or rarely talked to their husband about contraception (50%), were more likely to talk to a gynecologist about contraceptive methods. Moreover, women who intended to have children and resided in rich residential districts of Tehran were more likely to use the internet as their primary source of contraceptive knowledge. While gynecologist was the main source of contraceptive knowledge for older and more educated women, younger and less educated women, living in moderate to poor communities were more likely to gain their required contraceptive information from midwives in the public health centers. Finally, women whose primary source of contraceptive knowledge was their "social networks" were mostly young, educated, and unemployed, intended to have children, always talked to their husband easily about contraception and lived in moderate or poor residential districts.

**Table 2:** Percent distribution of Withdrawal users (married women aged 15–49 years) by key source of contraceptive information, according to selected characteristics: Tehran, Iran (n = 79).

Characteristics	Source of contraceptive information				n
	Gynecologist	Internet	Midwife in the Health Center	Social networks^a^	
All women	41.8	21.5	19.0	17.7	79
Age					
< 35	35.1	18.9	21.6	24.3	37
35 +	47.6	23.8	16.7	11.9	42
Years of schooling					
< 13	34.6	23.1	30.8	11.5	26
13 +	45.3	20.8	13.2	20.8	53
Employment status					
Unemployed	35.8	22.6	18.9	22.6	53
Employed	53.8	19.2	19.3	7.7	26
Fertility intention					
Intend to have children	35.5	25.8	16.1	22.6	31
Don't intend to have children	45.8	18.8	20.8	14.6	48
Talking to husband on method *					
Always	38.2	25.5	12.7	23.6	55
Sometimes/rarely/never	50.0	12.5	33.3	4.2	24
Residential district (Wealth)*					
Northern (Rich)	40.7	37.0	14.8	7.4	27
Eastern & Southern (moderate-poor)	42.3	13.5	21.2	23.1	52

^a^Include ' Relatives, friends, or colleagues'. Significant level of Chi-square test: *p ≤ *0.05.*

### Evaluation of contraceptive withdrawal

Participants were asked to name up to two most important "advantages and disadvantages" of the withdrawal method. As Table 3 shows, the most frequently cited advantages of withdrawal were "has no side effects" (39%), "easy to use" (38%), "increases sexual pleasure and intimacy" (37%), and "has no cost and is accessible" (18%). A woman quoted a summary of advantages of withdrawal: "The natural method [withdrawal] doesn't enter extra or harmful substances or items into my body. It isn't like a condom that always worries me not to be torn. It doesn't have the side effects of the device [IUD] and you don't need to be constantly careful not to lift a heavy item so that the device does not come out of your body, or you don't have to worry about spotting. The natural method is completely at our own hand, and we use it carefully [before ejaculation or the release of a pre-ejaculate fluid]." (Aged 34, married for 13 years, 12 years of schooling, 2 children). Other woman stated, "I feel good during sex, and I feel more intimacy with this method [withdrawal]." (Aged 32, married for 6 years, 16 years of schooling, 1 child).

**Table 3:** Percentage distribution of Withdrawal users (married women aged 15–49 years) by advantages and disadvantages of Withdrawal: Tehran, Iran (n = 79).

Advantages	Percent
Has no side effects of other (modern) contraceptive methods	39.2
Easy to use	38.0
Increases sexual pleasure and intimacy	36.7
Has no cost & is accessible	17.7
Effective and reliable method	6.3
Others (none, no opinion, don't know)	3.8

Disadvantages	
Its high risk of unwanted pregnancies	36.7
Having fear and anxiety of unwanted pregnancies	34.2
No disadvantages	20.3
Decreases sexual pleasure of myself and my husband	7.6
Risk of infection	7.6

Respondents could give more than one answer, so the total percentages exceed 100%.

As the most common disadvantages of withdrawal, respondents reported, "its high risk of unwanted pregnancies" and "fear and anxiety of unwanted pregnancies" 37% and 34% of the time, respectively. For example, a woman cited, "There is pregnancy stress in using the natural method [withdrawal]. We made a mistake with this method once, so I got pregnant and had to abort it." (Aged 32, married for 7 years, 18 years of schooling, 0 child). Another woman quoted, "You may suddenly become pregnant like my second child." (Aged 26, married for 7 years, 12 years of schooling, 2 children). Only 8% of participants reported that withdrawal "decreases sexual pleasure" and has "risk of infection". For instance, some women quoted: "The most important drawback is that you have to pull out at the peak of pleasure." (Aged 36, married for 3 years, 14 years of schooling, 1 child); "It is more of a flaw for a man, and it reduces his pleasure, and for me it also reduces the pleasure because I like the liquid to spill inside my body." (Aged 34, married for 12 years, 14 years of schooling, 3 children); "It causes infections on both sides." (Aged 44, married for 24 years, 16 years of schooling, 1 child). One-fourth of participants believed that withdrawal "has no disadvantages".

### Attitudes toward withdrawal method

**Figure 1:**
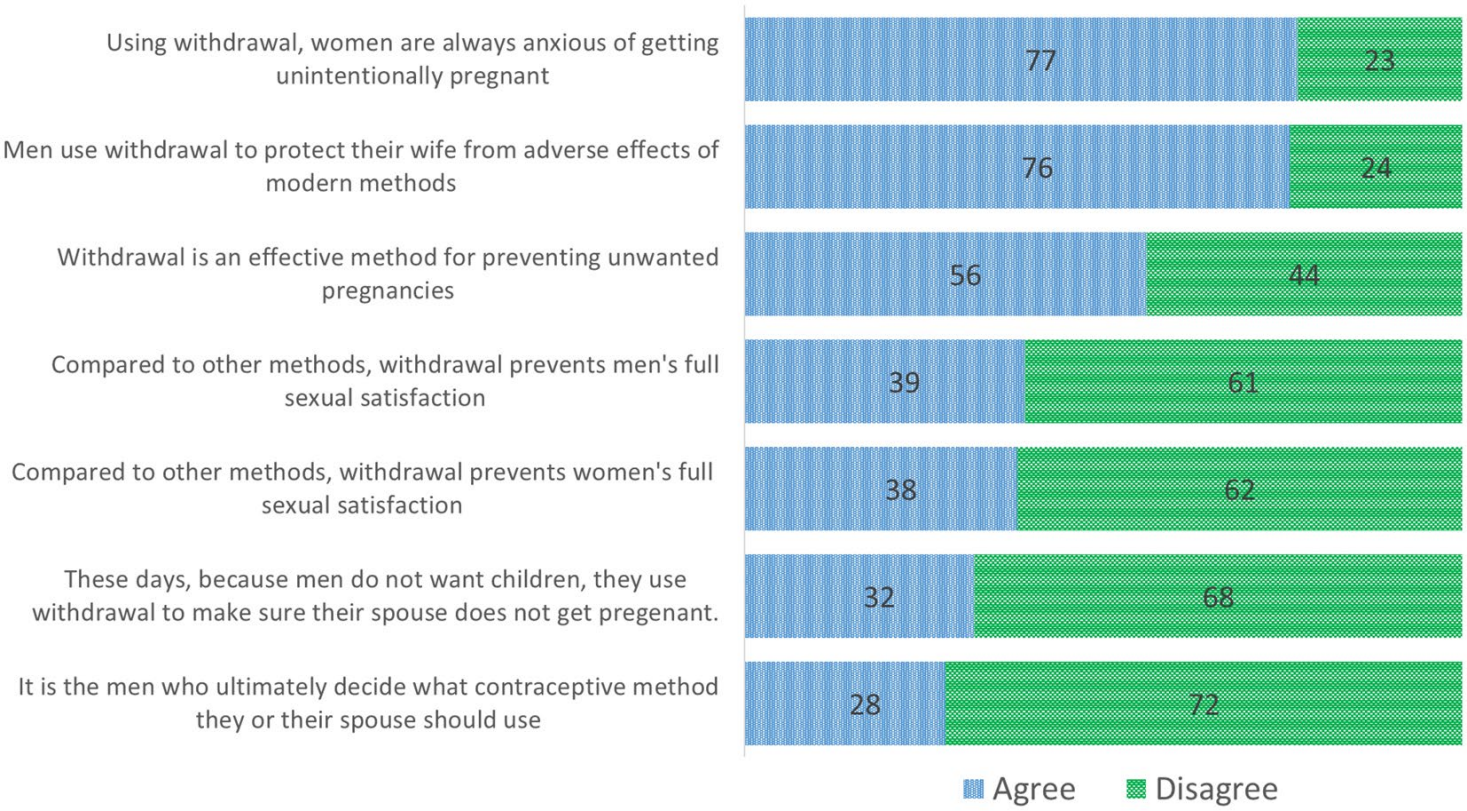
Percent destribution of withdrawal users (married women aged 15–49) by attitudes toward Withdrawal: Tehran, Iran 2021 (n = 79).

This study measured participants' attitudes toward different issues related to contraceptive withdrawal, including anxiety of unintended pregnancies, protecting women from adverse effects of modern contraceptive methods, effectiveness of withdrawal, decreased sexual satisfaction, and men's role in birth control and contraceptive decision-making. The results are presented in Fig. 1 and Table 4. The majority (77%) of withdrawal users agreed, "Women who are using withdrawal are always under the stress of getting unintentionally pregnant." The approval of this item was significantly higher among women aged under 35 versus 35 or older (86% vs. 69%), those who 'always' rather than 'sometimes or rarely' easily talk to their husband about contraception (84% vs. 63%), and among women who reported that withdrawal use was mostly their husband's preference rather than their own preference (100% vs. 60%).

**Table 4:** Attitudes toward Withdrawal among Withdrawal users (married women aged 15–49 years) by selected characteristics (%): Tehran, Iran (n = 79).

Characteristics	Using it, women are anxious to get pregnant		Men want to protect their wife's health		It is an effective method		It hinders men's full sexual gratification		It hinders wife's full sexual gratification		Using it, men want fully to control fertility		Men is the final contraceptive decision maker		n
	Agree	Disagree	Agree	Disagree	Agree	Disagree	Agree	Disagree	Agree	Disagree	Agree	Disagree	Agree	Disagree	
All Women	77.2	22.8	75.9	24.1	55.7	44.3	39.2	60.8	38.0	62.0	31.6	68.4	27.8	72.2	79
Age															
< 35	86.5	13.5	78.4	21.6	62.2	37.8	29.7	70.3	38.8	62.2	32.4	67.6	29.7	70.3	37
≥ 35	69.0	31.0	73.8	26.2	50.0	50.0	47.6	52.4	38.1	61.9	31.0	69.0	26.2	73.8	42
Chi-square (*P*-value)	**3.4(0.05)**		0.23 (0.64)		1.2 (0.28)		2.6 (0.10)		0.001 (0.98)		0.02 (0.88)		0.12 (0.73)		
Residential district (Wealth)															
North (Rich)	85.2	14.8	77.8	22.2	63.0	37.0	59.3	40.7	66.7	33.3	44.4	55.6	25.9	74.1	27
East/south (Moderate/poor)	73.1	26.9	75.0	25.0	51.9	48.1	28.8	71.2	23.1	76.9	25.0	75.0	28.8	71.2	52
Chi-square Test (*P*-value)	1.5 (0.22)		0.08 (0.78)		0.88 (0.34)		**6.9 (0.01)**		**14.3 (0.001)**		**3.1 (0.05)**		0.1 (0.78)		
Employment status															
Employed	76.9	23.1	73.1	26.9	57.7	42.3	50.0	50.0	34.6	65.4	15.4	84.6	26.9	73.1	26
Unemployed	77.4	22.6	77.4	22.6	54.7	54.3	34.0	66.0	39.6	60.4	39.6	60.4	28.3	71.7	53
Chi-square Test (*P*-value)	0.02 (0.97)		0.18 (0.68)		0.06 (0.80)		1.8 (0.17)		0.19 (0.67)		**4.7 (0.03)**		0.02 (0.90)		
Talking to husband on method															
Always	83.6	16.4	83.6	16.4	50.9	49.1	41.8	58.2	34.5	65.5	32.7	67.3	23.6	76.4	55
Sometimes/rarely/never	62.5	37.5	57.3	41.7	66.7	33.3	33.3	66.7	45.8	54.2	29.2	70.8	37.5	62.5	24
Chi-square Test (*P*-value)	**4.2 (0.04)**		**5.9 (0.02)**		1.7 (0.20)		0.51 (0.48)		0.90 (0.34)		0.10 (0.75)		1.6 (0.21)		
Who mostly prefers withdrawal?															
Wife	60.0	40.0	73.3	26.7	46.7	53.3	46.7	53.3	33.3	66.7	26.7	73.3	13.3	86.7	15
Husband	100.0	0.0	63.6	36.4	45.5	54.5	54.5	45.6	36.4	63.6	54.5	45.5	81.8	18.2	11
Both	77.4	22.6	79.2	20.8	60.4	39.6	34.0	66.0	39.6	60.4	28.3	71.7	20.8	79.2	53
Chi-square Test (*P*-value)	**5.8 (0.05)**		1.3 (0.53)		1.4 (0.49)		2.05 (0.36)		0.21 (0.90)		3.1 (0.21)		**18.8** **(0.001)**		

Significant values are in bold.

Most (75%) agreed that "Men use withdrawal to protect their wife from the side effects of modern methods". Women who "always" easily talked to their husband about contraceptive issues (84%) were significantly more likely to support this attitude. Regarding the effectiveness of withdrawal, only 56% agreed that it was an effective contraceptive method. This item was largely approved by those who were younger (62%), lived in the rich residential areas (62%), and together with their husband preferred to use withdrawal (60%). Moreover, about two-thirds of women disagreed that withdrawal would prevent men or women from reaching a "full sexual satisfaction." This mentality was significantly higher among women who were living in moderate-poorer versus rich residential areas (71% vs. 41% for men; 77% vs. 33% for women). Similarly, most respondents (68%) disagreed with the view that "since men do not want children, they control fertility themselves by withdrawal to make sure to prevent unintended pregnancies." Employed versus unemployed (85% vs. 60%), those living in moderate-poor versus rich residential areas (75% vs. 56%), and women who either themselves alone or together with their husband rather than their husband alone preferred to use withdrawal (73%, 72% vs. 46%), were significantly more likely to disagree this view. Finally, 72% of participants disagreed with the view that "men are the ultimate decision-makers for selecting contraceptive method". The proportion of women who significantly disagreed with this item was higher among those who either themselves alone or together with their husband rather than their husband alone preferred to use withdrawal (87%, 79% vs. 18%).

### Reasons for using contraceptive withdrawal

Table 5 shows the reasons given by participants for not using any modern methods instead of withdrawal, and specifically the reasons for not using male condoms instead of withdrawal. The most common reason given by 70% of women was "adverse effects of modern methods". For example, a woman reported, "I am using withdrawal because of the side effects of other methods that we all tried. I'm allergic to condoms. I was bleeding with IUDs. I had stomach bleeding with the pill. I was paralyzed in the body with the injection" (aged 33, married for 12 years, 14 years of schooling, 1 child). Another participant quoted: "I took the pill, but it gave me a high blood pressure. I took IUD twice, but it was expelled" (aged 40, married for 20 years, 12 years of schooling, 2 children). Other reason cited was "fear of the side effects" (24%), which was mostly based on what they heard from the experience of people around them. For instance, a woman quoted: "They [other people] say that condoms cause fibroids, and the pill make people nervous. They also say if you want to use IUDs, you should not do heavy work" (aged 29, married for 5 years, 16 years of schooling, 1 child).

**Table 5:** Percentage distribution of Withdrawal users (married women aged 15–49 years) by reasons for not using modern contraceptive methods: Tehran, Iran 2021 (n = 79).

	Percent
Reasons for not using any modern methods instead of withdrawal:	
Side effects of modern methods	69.6
Reduction of sexual pleasure	30.4
Fear of side effects of modern methods	24.1
Unable to use the method properly/modern methods are difficult to use	15.2
We are conformable with Withdrawal method/it is a reliable method	15.2
Because of my health situation	12.7
Don't trust modern methods (get pregnant)	7.6
Difficult to access modern methods	6.3
Recent delivery	6.3
Cost of modern methods	3.8

Reasons for not using condoms instead of withdrawal:	
Condoms reduce my husband's sexual pleasure	59.5
Health concerns (skin allergic to condoms and vaginal dryness)	16.5
Condoms reduce **our** sexual pleasure (husband & wife)	15.2
Socioeconomic difficulties in accessing condoms	7.6
Confidence in Withdrawal effectiveness vs. condoms	7.6
Husband's lack of competence in using condoms properly	6.3
Wife does not like condoms	5.3

*Respondents could give more than one reason, so the total percentages exceeded 100%.

Women also cited "reduction of sexual pleasure" and "difficulties in using modern methods properly" 30% and 15% of the time, respectively. Most quotations regarding reduction in sexual pleasure was related to condoms or IUDs. For example, a participant quoted: "My husband disagrees with the device [IUD] and the condom because he says he feels that an external factor prevents the sexual relationship" (aged 36, married for 12 years, 10 years of schooling, 2 children). Contrary to the general expectation, only 6% and 4% of women reported "access" to and "cost" were respectively the reasons for not using modern methods. The reasons given for difficulties in access to modern methods were mostly "the shame of purchasing condoms" and "methods are not being readily available". Moreover, 15% of participants reported, "they are comfortable with withdrawal and it is a reliable method".

As two male contraceptive methods, participants were asked to give reasons for not using male condoms rather than withdrawal. The results in Table 5 show that reduction in the sexual pleasure of husband or both husband and wife were the most common reasons cited 60% and 15% of the time. Most women quoted that "my husband does not want to exist a hindrance during intercourse and he wants the skin-to-skin relationship to be perfect." One-sixth (17%) of participants gave a health reason related to having skin allergies to condoms or vaginal dryness. Only 8% of respondents did not use condoms because of socioeconomic difficulties in accessing condoms. They mainly cited "because of the embarrassment of purchasing a condom" (6%), and partly quoted "it is expensive" (2%). Moreover, 7% of respondents preferred withdrawal in relative to condoms because they found it "more effective" and other respondents (6%) use withdrawal because their husband is "not able to use condoms properly".

Bivariate analysis in Table 6 shows that reported reasons for using contraceptive withdrawal significantly differ by the role of wife and husband in choosing withdrawal. The results show that 37% of all reasons reported are about "side effects of modern methods" followed by "fear of side effects of modern methods (16%), and "reduction in sexual satisfaction" (14%). The reason "side effects of modern methods" was given mostly by women who themselves alone versus their husband alone preferred to use withdrawal (52% vs. 19%). In addition, women whose husband alone preferred withdrawal to modern methods versus those who jointly with their husband chose withdrawal were more likely to cite "reduction in sexual pleasure" and "fear of side effects" (28% vs. 10%; 25% vs. 15%).

**Table 6:** Percent distribution of reasons for not using modern methods versus contraceptive withdrawal, by withdrawal users' selected characteristics: Tehran, Iran (n = 198 responses).

Women's characteristics	Reasons for not using modern methods vs. withdrawal				n
	Side effects of modern methods	Reduction in sexual satisfaction	Fear of side effects of modern methods	Others^a^	
All reasons	36.9	14.1	15.7	33.3	198
Age					
< 35	41.0	13.0	18.0	28.0	100
35 +	32.7	15.3	13.3	38.8	98
Chi-square Test (*P*-value) = 3.6 (0.31)					
Years of schooling					
< 13	30.3	16.7	21.2	31.8	66
13 +	40.2	12.9	12.9	34.1	132
Chi-square Test (*P*-value) = 3.6 (0.31)					
Number of pregnancies					
< 2	44.9	15.4	14.1	25.6	78
2	25.6	14.0	19.8	40.7	86
3 +	47.1	11.8	8.8	32.4	34
Chi-square Test (*P*-value) = 10.4 (0.09)					
Previous contraceptive method					
Withdrawal/none	35.3	15.0	12.0	37.6	133
Modern methods	40.0	12.3	23.1	24.6	65
Chi-square Test (*P*-value) = 6.1 (0.11)					
Talking to husband on methods					
Always	40.6	11.9	15.4	32.2	143
Sometimes/rarely/never	27.3	20.0	16.4	36.4	55
Chi-square Test (*P*-value) = 4.0 (0.26)					
Primary source of contraceptive knowledge					
Healthcare providers^b^	36.5	19.2	14.4	29.8	104
The internet	34.0	7.5	22.6	35.8	53
Social networks	41.5	9.8	9.8	39.0	41
Chi-square Test (*P*-value) = 8.1 (0.22)					
Who mostly prefers withdrawal?					
Wife	51.5	15.2	9.1	24.2	33
Husband	19.4	27.8	25.0	27.8	36
Both wife and husband	38.0	10.1	14.7	37.2	129
Chi-square Test (*P*-value) = 15.9 (0.01)					

The row percentages are based on row marginal total responses. Respondents could give more than one reason, so the total responses (n = 198) exceeded the number of women (n = 79). ^a^Include 'difficult to use modern methods', 'my health condition', 'being comfortable with withdrawal', and 'recent pregnancy'. ^b^Include 'gynecologist' and 'midwives in health units'.

Other bivariate results, though not statistically significant, show that "side effects of modern methods" were given mostly by women who refer primarily to their social networks for contraceptive information (42%), "always" easily talk to their husband about contraceptive issues (41%), used modern methods before withdrawal (40%), had some postsecondary education (40%) and aged less than 35 (41%). Furthermore, women who 'sometimes or rarely' easily talked to their husband about contraceptive issues, and primarily referred to healthcare providers for contraceptive information (19%), mostly gave the reasons "reduction in sexual pleasure"). The reason "fear of side effects" was given largely by women aged under 35 (18%), had some secondary or less education (21%), used modern methods prior to withdrawal use (23%), and utilized "the internet" as their main source of contraceptive information (23%).

### Willingness to switch to modern methods

Table 7 shows that 75% of withdrawal users would not be willing to switch to a modern contraceptive method, even if the modern methods were freely accessible, while the rest (25%) were willing to do this switch. The bivariate results indicate that women and their husbands with postsecondary education, those who did not use modern methods prior to withdrawal, and those who jointly with their husband or husband alone decided to use withdrawal are more likely not to be willing to switch to modern methods. Specifically, the odds of willingness to switch to modern methods were respectively 72% and 80% lower among women with postsecondary education and those whose husbands had postsecondary education than some secondary education. Participants who were using modern methods before current withdrawal use and women who themselves alone preferred to use withdrawal, were respectively 6.4 and 3.4 times more likely to be willing to switch to modern methods, if they would be provided freely and easily.

**Table 7:** Percent distribution and unadjusted odds ratios of switching to modern methods, if they are provided freely and easily, by selected covariates among Withdrawal users (married women aged 15–49 years): Tehran, Iran (n = 79).

Covariates	Will you switch to modern methods if they are provided to you freely and easily?		N	Odds ratio (95% CI)	*P*-value
	No (%)	Yes (%)			
All women	74.7	25.3	79	**–**	**–**
Women's years of schooling**					
≤ 12	57.7	42.3	26	1:00	
> 12	83.0	17.0	53	0.28 (0.10–0.80)	0.02
Husband's years of schooling***					
≤ 12	55.2	44.8	29	1.00	
> 12	86.0	14.0	50	0.20 (0.07–0.59)	0.004
Contraceptive method used prior to the withdrawal***					
None/the same withdrawal	83.6	16.4	61	1:00	
Modern methods	44.4	55.6	18	6.4 (2.02–20.15)	0.002
Who mostly prefers withdrawal?*					
Wife	53.3	46.7	15	3.4 (1.05–11.21)	0.04
Husband/Husband & wife	79.7	20.3	64	1:00	

Significant level of Chi-square test: *p ≤ 0.05; **p ≤ 0.01; ***p ≤ *0.001.*

## Discussion

This descriptive study examined the reasons for withdrawal use as a birth control method in a pilot survey of withdrawal users who referred to public health centers in the north, eastern and southeastern districts of Tehran. The reasons for choosing withdrawal were explored along with women's source of contraceptive knowledge, evaluation of and attitudes to withdrawal, which are assumed to affect couples' rationales for choosing withdrawal method and willingness to switch to modern methods.

According to the findings, the number one reason reported by the withdrawal users for using withdrawal instead of modern methods was the 'adverse effects of modern methods' (70%), followed by 'reduction in sexual pleasure' (30%), and the 'fear' of the side effects of modern methods (24%). Though similar findings have been reported by previous studies^29,34^, the current study for the first time found that in addition to women's health concerns, 'lack of competence in using modern methods properly' was the other reason for using withdrawal, reported by 15% of withdrawal users. The inability to use a contraceptive method properly or being afraid of the accompanying side effects can be due to lack of accurate contraceptive knowledge and counselling, normally acquired from professional healthcare providers. This is supported by the bivariate results indicating that the reasons 'difficulty in using modern methods' and 'fear' of their side effects were mostly given by women whose primary source of contraceptive knowledge were their "social networks" and 'the internet", respectively. Other studies also found that health behaviors, including contraceptive choice, are affected by information gained from the individual's social networks, including friends and family members, and the media^35–39^.

Moreover, the reasons for not using modern methods were also related to the role of wife and husband in preferring withdrawal to modern methods. While 'side effects of modern methods" was the reason given mostly by women who either themselves alone or with their husband together preferred to use withdrawal, 'reduction in sexual pleasure' and 'fear of side effects' were mostly reported by those whose husband was the sole decision maker in choosing withdrawal. In addition, primarily women who jointly with their husband preferred withdrawal to modern method gave the reasons of “difficulty in using modern methods properly” and “women's health condition”. These results suggest a greater spousal cooperation in contraceptive decision-making, especially when the couples feel the side effects of modern methods risk women's health and they are not able to use the modern method properly. This evidence supports "the notion that family planning is women’s business only is outdated"^40^. Conventionally, family planning programs encouraged women’s engagement in using modern contraceptive methods to improve their reproductive agency. However, as indicated by a recent study^41^, women’s engagement in the decision NOT to use contraceptive methods can also be an indicator of women's reproductive agency provided that the decision is based on the woman's choice, and for the achievement of self-determined reproductive goals^42^. The results of this study showed that women's engagement (alone or with their husband) in deciding "not to use" modern contraceptive methods for the sake of their health, or likely for addressing their incompetency in the proper use of modern methods demonstrates women's reproductive agency.

The "fear" and "health concerns" regarding the side effects of modern methods reported by withdrawal users cannot necessarily be the result of experiencing modern contraceptive methods. We found that only 23% of withdrawal users utilized a modern contraceptive method prior to withdrawal use. They switched either from condoms (mainly because of reduction in sexual pleasure), pills (mainly due to weight gain) or from IUD (largely due to bleeding and spotting) to withdrawal. An American study also found that both women and men who felt that condoms reduced their sexual pleasure were more likely to have used the withdrawal method^43^. The fear of the adverse effects of modern methods expressed by withdrawal users who have never used a modern method (77%), are most likely based on what they have "heard" from others or have "read" on the internet. For this reason, their source of contraceptive knowledge was examined. The results indicated that 40% of withdrawal users obtained their contraceptive knowledge and information primarily from the internet (22%), or their social networks including relatives, friends, and colleagues (18%). These women were more likely to intend to have children, while those whose primary source of contraceptive knowledge were healthcare professionals, namely gynecologists or midwives (60%), largely did not intend to have children. These findings suggest that women who are seriously thinking about spacing, or stopping childbearing are more likely to rely on the contraceptive knowledge acquired from a healthcare professional. While gynecologists and the internet were mostly the primary source of contraceptive information for older and more educated women, younger and unemployed women living in primarily moderate or poor communities referred to their social networks for their required contraceptive information. A qualitative study conducted in a poor community of Turkey also found that women often gathered their information about side effects of modern methods through their social networks, where the information was mainly based on rumours against the modern contraceptives and was biased^44^.

Among other reasons, the cost of modern contraceptive methods played a trivial role in choosing withdrawal over modern contraceptive methods. Specifically, the cost of modern methods was the last reason given by only 4% of withdrawal users for using withdrawal. Additionally, only 2% of withdrawal users expressed that they did not use condoms instead of withdrawal because of its costs. The other finding also showed that most withdrawal users (75%) would not be willing to switch to modern contraceptive methods, even if they were provided free of charge and being easily accessible. These results contrast with the argument that the use of withdrawal has increased because of the recent government's limitations on the provision of publicly funded family planning programs^45^. In fact, the commonly used contraceptives in Iran (i.e., condoms, pills and IUDs) are currently provided by private sectors with affordable prices^23^. Although the law has banned vasectomy, the Ministry of Health performs tubal ligation procedures (i.e., tubectomy) that are approved by a district-level Tubal Ligation Committee in the Ministry of Health. Moreover, if the Ministry recognizes that a pregnancy is likely to jeopardize woman’s life and her fetus, she will be entitled to receive free contraceptive services^23^.

On the other hand, 25% of withdrawal users were willing to switch from withdrawal to a modern method if the method were provided free of charge. These women and their husbands largely have low education, suggesting that women from lower socioeconomic status are more in-need of publicly funded family planning services. Other studies indicated that women who have a high, unmet need for modern contraceptive methods usually have a higher number of children, are at the end of their reproductive lives and want to stop childbearing^23,46^.

Women also evaluated withdrawal very positively in comparison with modern contraceptive methods. They believed that withdrawal has no side effects, is easy to use, and increases sexual pleasure and intimacy. Only 18% of withdrawal users pointed to the accessibility and economic advantage of withdrawal (i.e., "has no cost"). This evidence indicates that the preference of couples for withdrawal over modern methods is less to do with the costs and accessibility of modern contraceptive methods, especially after the recent limitations posed on the delivery of public family planning services in Iran. Overall, these findings imply that women view "withdrawal" as a healthier and sexually more pleasant contraceptive method, compared with modern methods. Though risk and fear of unwanted pregnancy were reported as the major disadvantage of withdrawal, women who had used withdrawal were more likely to be pleased with the pregnancy resulting from failure of withdrawal rather than modern contraceptive methods, and less likely to report the pregnancy as "unwanted"^15,43^.

The results regarding attitudes to withdrawal revealed that most withdrawal users, especially older women, agreed that they would be " anxious of getting unintentionally pregnant", when using withdrawal. Moreover, the majority of women, mostly those living in rich communities and those who "always" easily talked to their husband about contraceptive issues, approved their husband use of withdrawal to protect their health from adverse effects of modern contraceptive methods. Similarly, the findings of a qualitative study in Turkey showed that men used withdrawal to protect their wives from possible adverse effects of contraceptives^47^. Most women in our study, however, disapproved the views that withdrawal prevents full sexual satisfaction; men use withdrawal to have full control on fertility; and men are the ultimate contraceptive decision-maker. These results indicate a weak male dominance in contraceptive decision-making, which is contrary to the findings from neighboring countries, such as Pakistan in which the decision on contraception use is largely made by the husbands, especially those who opposed to using contraceptives due to religious reasons^48^.

Overall, this study demonstrated the evaluation, attitudes and concerns that women have about the use of modern contraceptive methods that normally require interacting with healthcare professionals to access. Women's beliefs and concerns about contraceptive methods affect subsequent contraceptive adoption, choice, and discontinuation^49^. So, program interventions addressing women’s contraceptive concerns and needs should address “the root causes” of women’s beliefs, attitudes, and evaluation of contraceptive methods, rather than call them “misconceptions”, “misinformation” or misperception”^50^. The results of this study support the statement that "there is no 'best' contraceptive, that families can be planned without recourse to outside assistance, and that efficient contraception need not be modern contraception."^3^.

## Conclusions

The findings of this study imply that couples resort to the traditional contraceptive method of withdrawal, not simply because of the cost of modern contraceptive methods, but mostly due to the "fears" of likely side effects of modern methods that they have heard about from people in their social networks or on the internet. The biased and exaggerated contraceptive knowledge shared between peers can be avoided by providing women with accurate contraceptive knowledge and counselling through professional healthcare providers. The study also cataloged the benefits women see in using withdrawal relative to modern methods. Their perceptions on contraceptive methods, especially withdrawal, are insightful and can provide healthcare providers with information about women's perspectives, which they may not have previously appreciated. The reasons for not using modern methods can be addressed by the healthcare providers during the family planning counseling, when women discuss the reasons for withdrawal use. The counselling needs to include follow-up visits in which women have the chance to discuss their concerns with using modern methods and to receive the counsellor's advice on how to deal with their likely side effects, or how to use withdrawal correctly and consistently. The findings help policy makers to evaluate and improve the existing "reproductive health" program.

## Limitations and future research

Similar to other studies, this research comes with some caveats. One limitation is the selectivity in the data. Since the study objective was to learn the reasons behind the use of withdrawal, only women reporting use of withdrawal at the time of the interview were included in this pilot study, and women who were using other contraceptive methods or discontinued using withdrawal were not included in this study. Therefore, this study does not reflect the perspectives and experiences of those women who switched from withdrawal to modern methods, and those who discontinued the use of withdrawal. A future investigation of the views of all contraceptive method users about withdrawal would clarify how unique the views of withdrawal users are. Another limitation is the small number of participants, since this study was a pilot survey conducted only in five health centers in the city of Tehran. Although the small number of participants can still meet the basic requirements for statistical analyses, the results are limited to withdrawal users in the districts in which the health centers are located, and hence cannot be generalized to the whole city of Tehran. In future research, an improved survey based on the results of this pilot study will be conducted in a representative sample of population living in the city of Tehran. In addition, it is recommended that the views of healthcare providers regarding the reasons for withdrawal use to be explored via a qualitative study. Finally, this study found significant gender differences (husband and wife) in the preference of withdrawal and reasons for not using modern methods. Future studies need to investigate men's rationales for choosing withdrawal and their concerns and beliefs about withdrawal.

## Data Availability

The datasets generated and analysed during the current study are not publicly available due to the data confidentiality agreement with Shahid Beheshti University of Medical Sciences but are available from the corresponding author on reasonable request.
